# The association between tuberculosis and the development of insulin resistance in adults with pulmonary tuberculosis in the Western sub-district of the Cape Metropole region, South Africa: a combined cross-sectional, cohort study

**DOI:** 10.1186/s12879-017-2657-5

**Published:** 2017-08-15

**Authors:** Lauren Philips, Janicke Visser, Daan Nel, Renée Blaauw

**Affiliations:** 1 Division of Human Nutrition, Faculty of Medicine and Health Sciences, Stellenbosch University, Cape Town, South Africa; 2 Centre for Statistical Consultation, Stellenbosch University, Cape Town, South Africa

**Keywords:** Pulmonary tuberculosis, Insulin resistance, Adults, HOMA-IR, QUICKI

## Abstract

**Background:**

The existence of a bi-directional relationship between tuberculosis (TB) and insulin resistance (IR)/diabetes has been alluded to in literature. Although diabetes has been linked to increased tuberculosis risk, the relationship between tuberculosis as a causative factor for IR remains unclear. The study aimed to determine if an association existed between tuberculosis and IR development in adults with newly diagnosed pulmonary tuberculosis at baseline. It was additionally aimed to document changes in IR status during TB follow-up periods.

**Methods:**

This cross-sectional study evaluated ambulatory participants at baseline for IR prevalence via anthropometry, biochemistry and diagnostic IR tests [homeostasis model assessment-IR (HOMA-IR) and quantitative insulin sensitivity check index (QUICKI)]. A prospective cohort sub-section study was additionally performed on approximately half of the baseline study population, who were followed-up at two and five months whilst on tuberculosis treatment. Summary statistics, correlation co-efficients and appropriate analysis of variance were used to describe and analyse data. Participants were excluded if they presented with other forms of tuberculosis, were HIV-positive, obese or had any pre-disposing IR conditions such as diabetes or metabolic syndrome.

**Results:**

Fifty-nine participants were included from August 2013 until December 2014 (33.95 ± 12.02 years old; 81.4% male). IR prevalence was 25.4% at baseline, determined by a calculated HOMA-IR cut-off point of 2.477. Patients with IR were younger (*p* = 0.04). Although the difference between IR levels in participants between baseline and follow-up was not significant, a decrease was observed over time. The majority of participants (61.0%) presented with a normal BMI at baseline. Mean baseline values of fasting glucose were within normal ranges (4.82 ± 0.80 mmol/L), whereas increased mean CRP levels (60.18 ± 50.92 mg/L) and decreased mean HDL-cholesterol levels (males: 0.94 ± 0.88 mmol/L; females: 1.14 ± 0.88 mmol/L) were found.

**Conclusions:**

The study found an association between tuberculosis and IR development in newly diagnosed pulmonary tuberculosis patients. Although not significant, IR levels decreased over time, which could be indicative of a clinical improvement. A high prevalence of IR amongst young tuberculosis patients therefore highlights the need for early identification in order to facilitate a reversal of IR and prevent possible IR-related complications.

## Background

The existence of a bi-directional relationship between tuberculosis (TB) and insulin resistance (IR)/diabetes has previously been alluded to in the literature. Although diabetes has been linked to an increased risk for TB development, the role of TB as a causative factor for development of IR remains unclear. Insulin resistance is often defined as a condition where the body cells become resistant to the effects of insulin [1–4], resulting in a larger than normal insulin release to maintain a normal glycaemic response in the body [5]. Insulin resistance has previously been postulated to have either a genetic or environmental causation [6] and has been implicated in the aetiology of several diseases/conditions, such as the metabolic syndrome, cardiovascular disease, polycystic ovarian syndrome, hypertension, type 2 diabetes mellitus and obesity [6–9]. Although accurate diagnosis and measurement of IR is currently challenging, the estimated global prevalence ranges from 20 to 40% in the general population [10, 11].

It has been hypothesised that stress resulting from a long-term infection, such as TB or HIV, could increase the occurrence of IR in the body [12–14]. Recent literature has also described occurrences of impaired glucose tolerance, distortions in carbohydrate metabolism and altered insulin action among newly diagnosed TB patients [15–17]. The pro-inflammatory response accompanying a period of infection is postulated to result in decreased insulin production, which leads to a hyperglycaemic state [12]. This process may also be accompanied by the release of certain stress hormones such as epinephrine, cortisol and glucagon, which further impair the action of insulin [13]. The phenomenon of ‘transient hyperglycaemia’ (subsiding of glucose intolerance upon diagnosis after active TB treatment) can also not be discounted [18, 19]. The effect of Rifampicin, one of the pharmaceutical agents used in the treatment of TB, has also been found to result in transient hyperglycaemia soon after treatment commencement due to its strengthening of intestinal glucose absorption [20]. Furthermore, according to Schwartz’s theories, it has been postulated that the pancreas could be assaulted by TB either via concomitant pancreatitis (resulting in heightened susceptibility to inflammation and/or amyloidosis) or via the forced habitation of the pancreas [21, 22]. Moreover, the persistence of TB bacteria in adipose tissue has been thought to be a possible causative factor for systemic IR [23].

To the author’s knowledge, there are no published studies documenting IR prevalence in pulmonary tuberculosis (PTB) patients using HOMA-IR and QUICKI tests. Given the high prevalence of both communicable and non-communicable diseases in South Africa, it was considered prudent to investigate the relationship between the two morbidities in a developing country setting. The study therefore aimed to determine if an association existed between TB and IR development in ambulatory adults with newly diagnosed PTB through the use of HOMA-IR and QUICKI. It was additionally aimed to document changes in IR status during follow-up. Once IR participants were identified, it was intended to document any differences between the IR and non-IR groups.

## Methods

### Study population

A descriptive, cross-sectional study was undertaken and participants were enrolled from the Albow Gardens clinic in the Western sub-district of the Cape Metropole region (South Africa) from August 2013 until December 2014. Recruitment was done via non-random, purposive sampling and participants were included in the study if they were newly diagnosed with PTB (either via molecular, microbiological or radiographical testing), were between the ages of 18–65 years old, on a standardised TB treatment regime, HIV-negative and willing to provide a blood sample. Participants were excluded if they presented with any other forms of tuberculosis, were HIV-positive, had increased anthropometrical values [obese (BMI ≥30 kg/m^2^) or increased waist circumference measurement (males >102 cm; females >88 cm)] or had any pre-disposing IR conditions (such as diabetes or metabolic syndrome). Once participants had been identified for participation in the cross-sectional study, approximately half (*n* = 29) were selected to be followed up at two and five months after tuberculosis treatment commencement by means of a prospective cohort sub-section study.

The study was approved by the Health Research Ethics Committee of the Faculty of Medicine and Health Sciences, Stellenbosch University in October 2012 (S12/08/227). Permission was also granted (April 2013) by the City of Cape Town (CoCT) to recruit participants from the Albow Gardens clinic (ref number:10346). Participants were required to give written informed consent prior to data collection. Privacy of participants for the duration of the study was ensured by making use of an anonymous approach.

### Anthropometry

The weight (calibrated, beam-balance scale) and height (fixed stadiometer) of each participant was measured at baseline according to standardised techniques [24]. The Body Mass Index (BMI) of each participant was subsequently calculated and classified according to the formula: *Weight (kg)/height (m)* ^*2*^ [25]. The waist and hip circumferences were measured using a non-stretchable tape measure and were also performed using standardised techniques [24]. The waist: hip ratio was calculated using the formula: *waist circumference (cm)/hip circumference (cm)* and classified according to the World Health Organisation (WHO) cut-off points [26]. Four skinfold measurements were taken, namely biceps, triceps, subscapular and suprailieac, according to standardised techniques for these measurements. A reliable skinfold calliper (Harpenden) was used to perform all measurements. Fat mass, fat free mass and body fat percentage were then calculated based on the sum of skinfold measurements and classified accordingly. The average of three measurements was taken for weight, height, waist and hip circumferences, as well as skinfold measurements. Only the height was measured at baseline, whilst all remaining anthropometrical measurements were performed at all follow-up visits.

### Biochemistry

A maximum of 15 ml of blood was collected from each participant, after a 10-h overnight fast. Samples were taken by a trained nursing sister at the data collection site and transported under correct storage conditions to the laboratory of the National Health Laboratory Services (NHLS). Albumin (bromocresol green solution), fasting glucose, C-reactive protein (CRP) and lipid profile were analysed using the Siemens Advia 1800. With regard to the lipid profile, total cholesterol was analysed using an enzymatic method, whilst the triglyceride value was calculated using the Fossati three-step enzymatic reaction. LDL-cholesterol was determined by means of the Friedewald formula [27]. The white cell count was performed by the Siemens Advia 2120 whilst fasting insulin was analysed with the help of ADVIA Centaur® Insulin Lite Reagent and Solid Phase. Standardised reference ranges of the NHLS were used to classify biochemical values.

### Diagnostic IR-tests

The HOMA-IR diagnostic test was performed according to the following formula: [*Fasting serum insulin (*μU*/ml) x fasting plasma glucose (mmol/L)*]/22.5 [28]. The QUICKI measurement is the inverse logarithm of the HOMA-IR calculation, namely: *1/* [*log (fasting insulin* μU*/ml) + log (fasting glucose mg/dL*] [29]. As there is currently no standardised cut-off point for either the HOMA-IR or the QUICKI measurement, the data generated by this study was used to calculate a relevant HOMA-IR cut-off point. This was based on the lower limit of the upper quartile (P75), as has been performed in similar studies [30–33]. Once the HOMA-IR cut-off had been calculated, the corresponding QUICKI value was determined by means of a receiver operating characteristic (ROC) curve analysis. Any individual having a HOMA-IR value greater than the calculated cut-off point, as well as below the QUICKI cut-off point, was classified as having possible IR. The HOMA-IR was used as the primary tool for identifying IR and the QUICKI was calculated to reinforce or compare results.

### Statistical analysis

Statistical analysis was performed using STATISTICA version 12 [StatSoft Inc. (2014)] and Microsoft Excel 2010. Summary statistics were used to describe data. Results were expressed as mean ± standard deviation. Regression and correlation analysis were utilised to document the relationship between continuous variables. Appropriate analysis of variance was used to investigate relations between continuous variables and nominal variables, whilst Bootstrap procedures were used in cases where the residuals were not normally distributed. For variables measured repeatedly over time, repeated measures ANOVA were done with the compound symmetry assumption on the correlation structure over time. ROC curves were performed to calculate the corresponding QUICKI cut-off point at baseline. A *p*-value of *p* < 0.05 represented statistical significance in hypothesis testing and 95% confidence intervals were used to describe the estimation of unknown parameters. The power analysis was done to estimate the sample size of participants needed at baseline (*n* = 30) and those seen at two and five months (*n* = 29). A power of 90% was able to detect a medium effect size of 0.6.

## Results

**Fig. 1:**
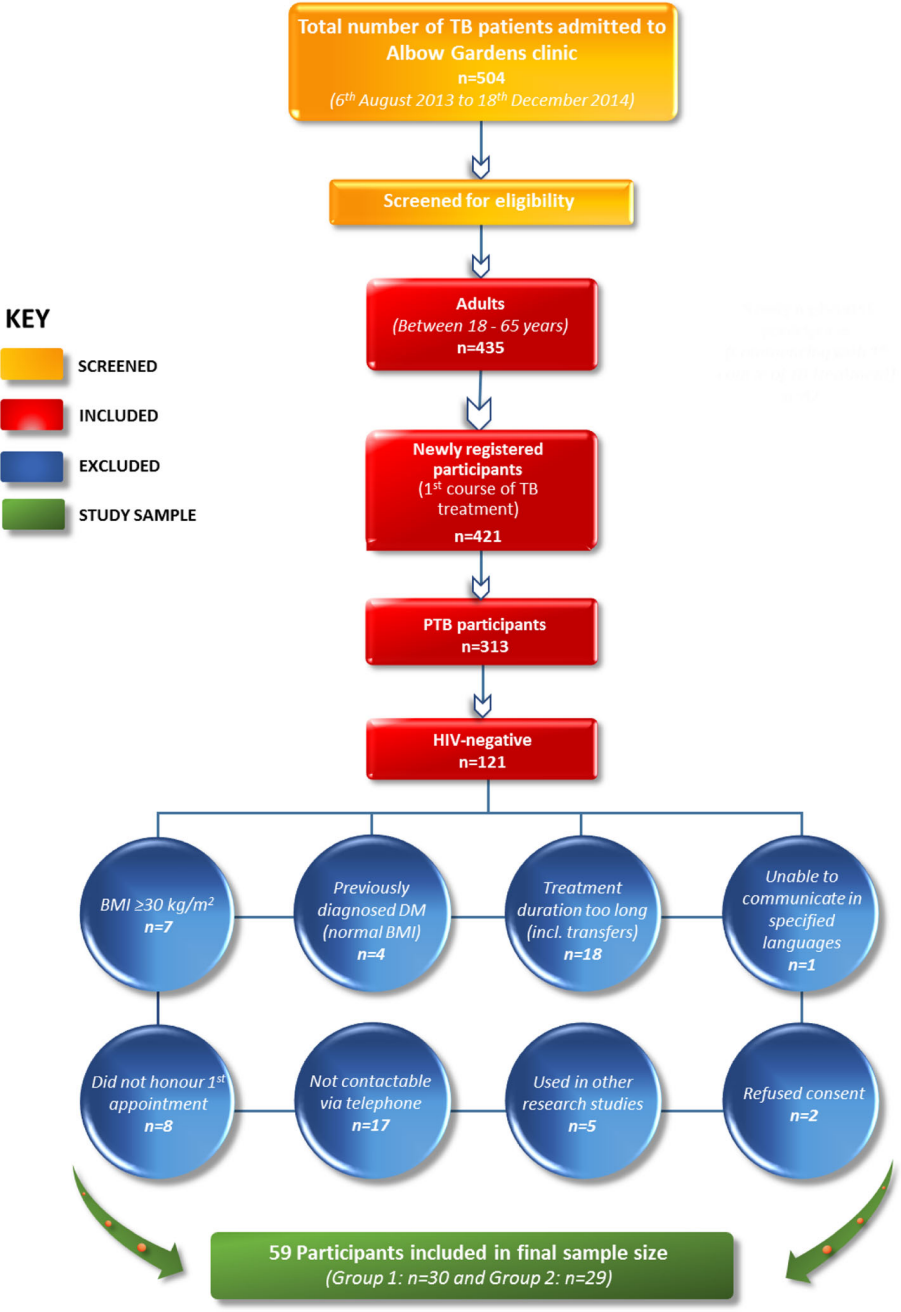
Flow diagram for inclusion of study participants. Group 1 = Participants seen at baseline only; Group 2 = Participants seen at baseline, two and five months; TB = tuberculosis; PTB = pulmonary tuberculosis; HIV = human immunodeficiency virus; BMI = Body Mass Index; DM = diabetes mellitus

Fifty-nine participants were recruited during the 17 month data collection period. A flow diagram indicating the inclusion of study participants is shown in Fig. 1. The majority of patients were male (81.4%) and had a mean age of 33.95 ± 12.02 years.

### Anthropometry

The majority of participants (*n* = 36, 61.0%) had a baseline BMI in the normal range according to WHO cut-off points (18.50–24.99 kg/m^2^) [25], while 33.9% (*n* = 20) had a BMI of <18.5 kg/m^2^, classifying them with varying levels of underweight. All of the anthropometrical measurements experienced a significant increase over time (*p* < 0.05), except for the biceps skinfold (Table 1). These increases occurred mainly in the first two months after treatment commencement.

**Table 1:** Changes in anthropometrical and biochemical variables over time with repeated measures ANOVA in follow-up group (*n* = 29)

Variable	Unit	ANOVA (F-test)	Baseline Mean (SD)	Two months Mean (SD)	Five months Mean (SD)	*p*-value
**Anthropometry**						
**Weight**	**kg**	**F(2,56) = 39.51**	**56.83 (10.66)**	**59.76 (11.65)**	**61.37 (11.26)**	**<0.001**
**BMI**	**kg/m** ^**2**^	**F(2,56) = 39.34**	**19.65 (2.62)**	**20.66 (2.93)**	**21.21 (2.68)**	**<0.001**
**Waist circumference**	**cm**	**F(2,56) = 38.44**	**70.38 (8.84)**	**72.95 (9.32)**	**75.40 (9.18)**	**<0.001**
**Waist:hip ratio**	**-**	**F(2,56) = 36.29**	**0.83 (0.08)**	**0.85 (0.09)**	**0.88 (0.09)**	**<0.001**
Biceps skinfold	mm	F(2,56) = 1.81	3.16 (1.45)	3.36 (1.96)	3.49 (2.02)	0.174
**Triceps skinfold**	**mm**	**F(2,56) = 10.05**	**7.01 (4.05)**	**7.49 (4.29)**	**8.13 (4.63)**	**<0.001**
**Subscapular skinfold**	**mm**	**F(2,56) = 10.22**	**7.77 (2.85)**	**8.26 (2.69)**	**8.79 (3.35)**	**<0.001**
**Suprailiac skinfold**	**mm**	**F(2,56) = 6.81**	**6.10 (4.61)**	**7.32 (5.50)**	**7.57 (5.11)**	**0.002**
**Sum of skinfolds**	**mm**	**F(2,56) = 8.64**	**24.03 (11.79)**	**26.43 (13.47)**	**27.99 (14.18)**	**<0.001**
**Fat mass**	**kg**	**F(2,56) = 17.63**	**7.22 (4.34)**	**8.38 (4.39)**	**8.73 (4.50)**	**<0.001**
**Fat free mass**	**kg**	**F(2,56) = 38.11**	**49.61 (9.56)**	**51.38 (10.13)**	**52.63 (10.23)**	**<0.001**
**Percentage body fat**	**%**	**F(2,56) = 10.89**	**12.58 (6.53)**	**13.85 (5.96)**	**14.19 (6.31)**	**<0.001**
**Biochemistry**						
**Albumin**	**g/L**	**F(2,56) = 15.36**	**39.97 (4.06)**	**42.55 (3.90)**	**43.41 (3.77)**	**<0.001**
Fasting glucose	mmol/L	F(2,56) = 2.11	4.78 (0.61)	4.83 (0.58)	4.57 (0.50)	0.131
**CRP**	**mmol/L**	**F(2,56) = 21.63**	**56.72 (50.83)**	**27.54 (32.11)**	**12.48 (15.07)**	**<0.001**
Fasting insulin	mU/l	F(2,56) = 1.16	14.62 (25.98)	11.91 (15.81)	8.99 (6.77)	0.320
**Total cholesterol**	**mg/L**	**F(2,56) = 10.43**	**3.55 (0.95)**	**4.20 (1.06)**	**3.94 (0.84)**	**<0.001**
Triglycerides	mmol/L	F(2,56) = 0.85	0.85 (0.30)	0.93 (0.38)	0.89 (0.38)	0.432
**HDL-cholesterol**	**mmol/L**	**F(2,56) = 14.50**	**0.99 (0.30)**	**1.30 (0.46)**	**1.31 (0.38)**	**<0.001**
**LDL-cholesterol**	**mmol/L**	**F(2,56) = 4.97**	**2.17 (0.75)**	**2.48 (0.87)**	**2.22 (0.70)**	**0.010**
**White cell count**	**10ˆ9/L**	**F(2,56) = 16.30**	**8.74 (4.18)**	**6.98 (2.91)**	**5.92 (2.01)**	**<0.001**

*SD* = standard deviation, *BMI* = Body Mass Index, *CRP* = C-reactive protein, *HDL* = high density lipoprotein, *LDL* = low density lipoprotein

Bold variables indicate statistical significance

### Biochemistry

Baseline biochemistry results showed that the majority of patients (*n* = 50; 84.7%) had an increased mean CRP level (60.18 ± 50.92 mg/L) and decreased mean HDL-cholesterol level (*n* = 41; 69.5%) at baseline (males: 0.94 ± 0.88 mmol/L; females: 1.14 ± 0.88 mmol/L). Mean fasting glucose (4.82 ± 0.80 mmol/L), fasting insulin (11.37 ± 19.20 mU/L), albumin (39.32 ± 4.35 g/L) and white cell count (8.84 ± 3.56 10ˆ9/L) were all in the normal reference ranges. According to fasting glucose levels at baseline, 1.7% (*n* = 1) and 3.4% (*n* = 2) of participants could be classified as having impaired fasting glucose (IFG) (6.1 mmol/L – 6.9 mmol/L) and diabetes (≥7 mmol/L) respectively [34].

Significant changes over time were seen with a decrease in both CRP (<0.001) and white cell count levels (<0.001), while albumin experienced an increase (<0.001). Total, LDL and HDL-cholesterol experienced a significant increase in the first two months of treatment, followed by a tapering off between two and five months (Table 1).

### Insulin resistance

**Fig. 2:**
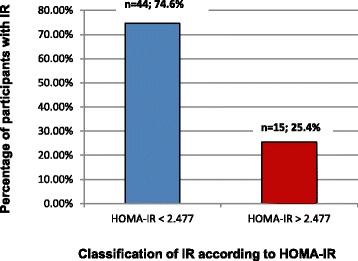
Classification of total population at baseline according to determined HOMA-IR cut-off point (*n* = 59). IR = insulin resistance; HOMA-IR = homeostasis model assessment-insulin resistance

The 75th percentile of the HOMA-IR was calculated using data collected from participants (cut-off value of 2.477). A corresponding QUICKI cut-off point was calculated on the basis of the HOMA-IR and a value of 0.336 was obtained. Using this information, 25.4% (*n* = 15) participants were classified with IR at baseline (Fig. 2). Participants with post-recruitment possible metabolic syndrome [according to International Diabetes Federation (IDF) (*n* = 2) [2, 35] and Adult Treatment Panel III (ATP III) (*n* = 1) [36] criteria] and diabetes (*n* = 2) were excluded in further sub-analyses, which resulted in a minimal difference in overall prevalence rates of IR (IDF: 24.6%; ATP III: 25.9%; possible diabetes: 24.6%).

**Fig. 3:**
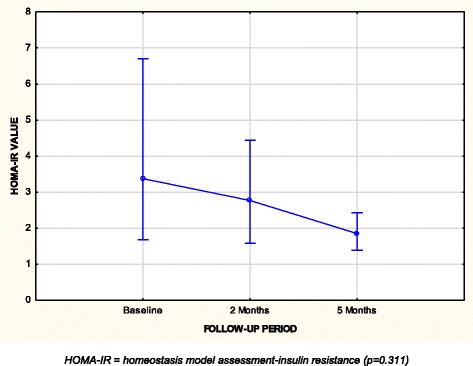
Changes in HOMA-IR values of participants over five-month follow-up period (*n* = 29). HOMA-IR = homeostasis model assessment-insulin resistance

Regression analysis showed the anthropometrical measurements of waist circumference (*r* = 0.507; *p* = 0.011), sum of skinfolds (*r* = 0.353; *p* = 0.039) and fat mass (*r* = −2.419; *p* = 0.08) were the best predictors of IR at baseline. Although the difference experienced between HOMA-IR levels in participants between baseline and follow-up periods was not significant (*p* = 0.311), a decrease was experienced over time (Fig. 3). Participants in the follow-up group (*n* = 29), showed a 31% prevalence of IR at baseline (*n* = 9), compared to 27.6% (*n* = 8) and 20.7% (*n* = 6) at two and five months respectively.

### Insulin resistant vs. non-insulin resistant groups

**Fig. 4:**
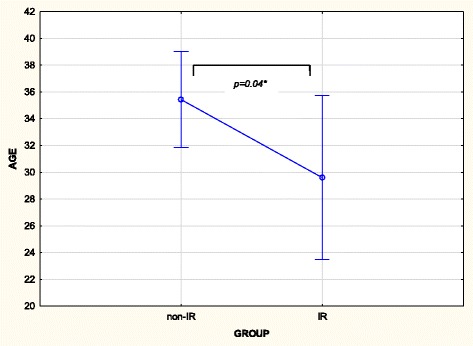
Comparison between the IR and non-IR group vs. age of participants (*n* = 59). IR: insulin resistance. *p* = 0.04*. Mean (years) ± SD: non-IR-group (35.43 ± 12.02); IR group (29.60 ± 11.29)

Patients with IR were shown to be younger (*p* = 0.04) (Fig. 4) and had a higher fasting insulin measurement (*p* < 0.01). Despite no significance between the groups in terms of CRP levels, the IR group had a lower mean value (*p* = 0.51). There were also no significant differences between gender, age groups, race, BMI classification or waist circumference classification between IR and non-IR groups at baseline (Table 2). Upon consideration of differences between IR and non-IR groups at the two and five month follow-up visits, there were no significant results for any of the variables.

**Table 2:** Comparison of baseline variables of IR group vs. non-IR group (continuous and categorical variables) (*n* = 59)

Variable (Baseline)	Unit	Non-IR Group (*n* = 44) Mean (SD)	IR Group (*n* = 15) Mean (SD)	*p*-value*
**Continuous variables**				
**Age**	**years**	**35.43 (12.02)**	**29.60 (11.29)**	**0.04**
Systolic blood pressure	mmHg	119.23 (17.78)	125.13 (11.57)	0.13
Diastolic blood pressure	mmHg	75.45 (12.46)	79.93 (10.42)	0.05
Albumin	g/L	38.75 (4.58)	41.00 (3.16)	0.17
Fasting glucose	mmol/L	4.71 (0.69)	5.13 (1.02)	0.16
CRP	mmol/L	64.10 (53.82)	48.91 (41.01)	0.51
**Fasting insulin**	**mU/l**	**5.63 (2.43)**	**28.20 (33.21)**	**<0.01**
Total cholesterol	mg/L	3.39 (0.87)	3.71 (0.95)	0.30
Triglycerides	mmol/L	0.88 (0.35)	0.95 (0.27)	0.38
HDL-cholesterol	mmol/L	0.99 (0.33)	0.93 (0.26)	0.56
LDL-cholesterol	mmol/L	1.98 (0.65)	2.35 (0.80)	0.24
White cell count	10ˆ9/l	8.79 (3.68)	8.99 (3.30)	0.68
**HOMA-IR**	**-**	**1.18 (0.54)**	**7.22 (10.32)**	**<0.01**
**QUICKI**	**-**	**0.39 (0.04)**	**0.31 (0.03)**	**<0.01**
		**Categorical variables**		
		**Groups assessed**		***p*** -value***
Gender		IR vs. non-IR		0.877
Age groups**		IR vs. non-IR		0.210
Race		IR vs. non-IR		0.816
BMI classification		IR vs. non-IR		0.217
Waist circumference classification		IR vs. non-IR		0.122

*SD* = standard deviation, *CRP* = C-reactive protein, *HDL* = high density lipoprotein, *LDL* = low density lipoprotein, *HOMA-IR* = homeostasis model assessment-insulin resistance, *QUICKI* = quantitative insulin sensitivity check index

Bold and shaded variables indicate statistical significance

*Mann-Whitney U test

**Age groups concerned: 18–30 years; 31–45 years; 46–65 years

*** *cc* Maximum-likelihood chi square test

## Discussion

Despite the mean BMI at baseline being classified in the normal range for both males and females, there was an overall prevalence of 33.9% of undernutrition (BMI <18.5 kg/m^2^) among the study population. These rates of undernutrition are in agreement with previously described rates of between 20% and 71.6% [37].

A low BMI at diagnosis has been linked to an increased risk of relapse [38] and demonstrates an indirectly proportional relationship existing between BMI and mortality risk [39]. Previous studies have also indicated that the occurrence of undernutrition among TB patients cannot be solely attributed to the disease itself but rather to a multitude of contributing factors, such as extreme poverty, food insecurity and reduced health-seeking behaviour [40, 41].

The majority of anthropometrical measurements showed a significant increase whilst the patients were on treatment, with the greatest changes taking place during the intensive phase (first two months of treatment) [42]. However, some patients failed to gain more than 5% weight over the five-month period and/or remained in the underweight BMI category, thereby placing them at greater risk of treatment failure and/or relapse at a later stage [38]. Overall weight gain of TB patients whilst on treatment is a common phenomenon [43–47] although some researchers are hesitant to use weight gain as a marker of successful response to treatment [48, 49].

Findings verifying low levels of albumin and high CRP levels in TB patients have been well documented in literature [50–54]. Baseline study results yielded an elevated mean CRP, while the mean albumin level was in the normal range. It is perhaps the raised CRP level that is more indicative of active TB disease because it has been described as a non-specific measure of systemic inflammation in the body [51]. The degree of CRP escalation has also been linked to the presence of weight loss and to disease severity, and this may increase levels even further [51]. The albumin levels were possibly not as severely affected due to the patients being relatively ‘well’ TB patients (i.e. with predominantly normal BMI’s, not hospitalised, drug-resistant or HIV-co-infected).

A lowered total cholesterol level has previously been described in the literature [51, 55, 56], in which it was hypothesised that prolonged persistence of the TB bacterium may result in cholesterol breakdown, especially in persons who had been infected latently for a period of time. Reduced HDL levels have been reported in numerous studies with various infectious or inflammatory conditions [57–59]. A study by Deniz et al. in 2007 showed lowered levels of HDL-cholesterol in their PTB-specific study population [56], which could be linked to the activated acute phase response (APR) often seen in TB disease [60–63].

The current study saw an increase in albumin levels, as well as a decrease in CRP (both over time). This would suggest a ‘resolution’ of the APR or a suppression of the inflammatory response [52], which is a phenomenon well reported in literature, both for albumin [64] and CRP [52–54, 64, 65]. There were, however, some patients in the follow-up group who still presented with a raised CRP at the five-month mark, which could perhaps indicate a sub-optimal treatment response [53] or the presence of other infections, especially if the CRP did not show an overall downward trend. Total cholesterol, as well as LDL-and HDL-cholesterol, experienced a significant increase during the intensive treatment phase. There were however no significant differences between the two-and five-month periods. This is an unusual finding because one might expect the values to increase even more at the end of the follow-up period and warrants more investigation given the important role of adequate cholesterol levels in protection against the mycobacteria.

One individual in the current study was classified with IFG (1.7%) and two with diabetes (3.4%). A systematic review published by Jeon et al. in 2010 showed total DM prevalence in TB patients to fall between the range of 1.9% and 35% [66], but some of these were diagnosed only after development of TB. The two ‘suspected’ diabetic patients were, however, not confirmed as having diabetes. In a cross-sectional study conducted in West Africa, participants displayed a 5% IFG and a 1.9% DM rate, which is comparable with the current study despite differing inclusion criteria [67]. A retrospective study performed in Sri Lanka found 7.1% pre-existing DM among their study sample, as well as 20% IFG and 2% DM rates (although lower fasting glucose cut-off values were used) [68]. These raised levels could also be attributed to the “stress/transient hyperglycaemia” phenomenon [18, 19, 69, 70], as well as the pro-inflammatory response [12, 13] and medication effects [20].

The HOMA-IR is one of the so-called ‘fasting indices’, which utilises fasting measurements of both glucose and insulin. The HOMA-IR has been used extensively in epidemiological research and as a routine measurement in clinical practice [71] and together with the QUICKI [29] has proved to be a very popular fasting index [28]. Although the current gold-standard method of diagnosing IR, the hyperinsulinaemic-euglycemic clamp (HEC), is very often the preferred diagnostic tool, the inherent study design did not lend itself to this particular technique, largely due to the time-consuming nature of the test and the fact that study participants were ambulatory out-patients. A notable drawback of the HOMA-IR and QUICKI is, however, the lack of a standardised cut-off point to identify individuals with IR. Previous studies have suggested that IR occurs between the HOMA-IR levels of 2.1 and 3.8 [28, 72], which is a vast range but in which the value of 2.477 calculated in this study fits comfortably. Using this calculated cut-off point, the IR prevalence at baseline among this study population was 25.4%, which equates to one in four persons with newly diagnosed PTB having IR. These findings were echoed by the QUICKI. This prevalence rate falls within the proposed range of 20–40% IR in the general population, of whom the majority are healthy persons (i.e. do not have TB) [11, 71]. Recent studies have duly reported high levels of hyperglycaemia, impaired glucose tolerance and DM in TB patients [12, 15–19, 66, 69, 70, 73–78].

The follow-up profiles of both HOMA-IR and QUICKI seemed to indicate an improvement in IR over time. Although there were no significant differences noted between any of the time frames, there was an overall downward pattern with the HOMA-IR and an upward curve for the QUICKI, both signifying a ‘lessening’ of the IR state. This ties in closely with an improved and diminishing inflammatory response, as demonstrated with the improved CRP, albumin and white cell count. This could also be due to the well-described ‘stress or transient’ hyperglycaemia seen to occur in the early stages after TB diagnosis and which often resolves with progression of treatment [18, 19, 73]. Despite the largely improved IR status over the five-month follow-up period, two participants who had normal HOMA-IR and QUICKI values at baseline subsequently developed IR at two and five months, and two patients redeveloped IR at five months after originally presenting with it at baseline. This contests the stress hyperglyacemia hypothesis, as well as the improvement of an inflammatory state, and is an interesting phenomenon to be pursued.

The development of IR has often been linked to the process of aging [29, 79], since age progression is generally associated with greater gains in body weight and/or fat mass, especially in the central area of the body [80], as well as with an increased prevalence of chronic diseases of the lifestyle, including metabolic syndrome [81, 82]. As the younger patients in this study displayed a greater prevalence of IR, this could signal the need for increased awareness of greater disease potential among the more youthful TB population, despite contrasting results to date. Recent research has indicated the possibility of genetic influences on the development of IR, although at the present moment, minimal variants have been associated herewith [83].

There is generally a greater risk of IR with increasing anthropometrical measurements [84], and positive correlations have been documented between IR and BMI [85, 86], waist and hip circumference, body fat content and weight gain [87, 88]. Several of these were seen in the current study (i.e. percentage body fat) and could perhaps be indicative of the phenomenon of increasing anthropometric measurements. A study conducted in 2012 by Addo et al. among adolescents in the United States showed that skinfolds (tricep and subscapular) were able to identify individuals at risk of developing IR [89]. The sum of skinfolds could perhaps be used as a marker to identify those at risk of developing IR, which may be prudent in populations that do not exhibit typical metabolic syndrome-like characteristics.

Because IR is largely termed an ‘inflammatory’ condition, levels of CRP (a positive acute phase protein) are often raised [90]. The inflammation typically found in IR is more of a ‘low-grade’ inflammatory state, which is often the result of the production of cytokines from visceral adipose tissue (VAT) [91]. The CRP levels are often produced as a response to the release of various cytokines designed to enable the inflammatory response [92]. One might thus have expected the CRP levels of the IR participants to be higher than the non-IR group, given the inflammatory nature of IR itself, its relationship with the APR and the fact that TB patients generally have increased CRP levels. However, the converse was seen. Given the fact that patients included in this study did not fit the conventional ‘metabolic syndrome’ or obesity profile, it could be speculated that they may have slightly higher levels of subcutaneous fat, compared with visceral tissue, which would, therefore, render a slightly less inflammatory profile, and this could have an inhibitory effect on CRP levels in the blood.

Leptin, an adipokine, has recently come under scrutiny regarding its role as a regulator of the immune system, as well as its effect on appetite [93] and IR reduction [94]. Findings regarding leptin levels in TB have to date been conflicting, with some studies showing higher leptin levels in TB patients [95–97] and others showing lower levels [98–101]. Individuals with reduced leptin concentrations have experienced an increased body weight over a short time duration, [102] which may be prudent in further investigating the link between TB and IR.

Recommendations for clinical practice include the implementation of integrative bi-directional screening (TB and DM) in health care facilities, as well as close monitoring of TB patients presenting with hyperglycaemia upon treatment commencement. Vulnerable patients with an undesirable anthropometrical, biochemical or clinical presentation should also be referred timeously for nutritional support, preferably during the intensive phase of treatment. Regarding future research avenues, it would be prudent to assess IR prevalence in other susceptible TB populations (HIV-co-infected, extra-pulmonary patients and different life stages) as well as to utilise additional IR diagnostic tools. Re-assessment of patients who developed IR at the five-month mark may also yield valuable results once treatment is completed. Although it was not possible in this study, a desired outcome could be the determination of a specific biomarker cut-off (e.g. CRP) that would be able to identify IR in TB patients and largely act as a diagnostic tool.

Limitations of the study included the paucity of standardised reference values for HOMA-IR and QUICKI, as well as issues surrounding the reliability of the fasting insulin measurement. It was furthermore unclear whether patients presented with hyperglycaemia before study recruitment. Logistical issues such as the stipulated time of data collection (as IR is reportedly higher in the morning), patients already having commenced with TB treatment, use of a single recruitment site, compliance with overnight fasting guidelines and inherent aspects of the study design (including sample size) could also be viewed as limitations.

## Conclusion

This study found an association between TB and IR development in newly diagnosed PTB patients, with one in four patients having IR. This high prevalence rate in the study population signals the need for early identification, especially in the younger and more vulnerable TB population, in order to facilitate a possible reversal of IR and prevent future IR-related complications.

## Abbreviations

APR: Acute phase response
ATP III: Adult Treatment Panel III
BMI: Body Mass Index
CoCT: City of Cape Town
CRP: C-reactive protein
DM: Diabetes mellitus
HDL: High density lipoprotein
HEC: Hyperinsulinaemic-euglycemic clamp
HIV: Human immunodeficiency virus
HOMA-IR: Homeostasis model assessment-insulin resistance
IDF: International Diabetes Federation
IFG: Impaired fasting
IR: Insulin resistance
LDL: Low density lipoprotein
NHLS: National Health Laboratory Services
PTB: Pulmonary tuberculosis
QUICKI: Quantitative insulin sensitivity check index
ROC: Receiver operating characteristic
SD: Standard deviation
TB: Tuberculosis
VAT: Visceral adipose tissue
WHO: World Health Organisation
